# Rotary Electromechanical System Integrating Non‐Reciprocal Memory and Combinational Logic

**DOI:** 10.1002/advs.202522133

**Published:** 2026-01-22

**Authors:** Shujia Chen, Don Straney, Damiano Pasini

**Affiliations:** ^1^ Department of Mechanical Engineering McGill University Montreal Canada

**Keywords:** electromechanical system, non‐volatile mechanical computing, rotation‐driven bistable mechanism, sequential logic

## Abstract

Recent advances in mechanical computing have harnessed bistable mechanisms with intrinsic memory to extend the scope of physically embodied intelligence, enabling history‐dependent behavior. However, existing mechanical computing architectures largely fail to integrate mechanically encoded memory with the logic operations necessary for sequential information processing, a foundational requirement for advanced computational tasks, such as autonomous sequential decision‐making. Here, we introduce a rotary electromechanical computing system that unifies non‐reciprocal mechanical memory with combinational logic, enabling reprogrammable sequential decision‐making within a single finite‐state‐machine (FSM) framework. The architecture consists of serially coupled rotary bistable units that collectively produce a history‐dependent, non‐reciprocal mechanical memory. The discrete geometric orientation of each unit encodes a binary state, which is transduced through a conductive network to execute logic operations. Under applied torque, the stacked system functions as a four‐bit FSM, in which state‐transition rules can be reconfigured by modifying the electromechanical coupling. This rotary FSM demonstrates multiple computing functionalities, including digital combination locking, in‐memory electromechanical computation, and reprogrammable digital control. By embedding non‐reciprocal state evolution and electrical logic directly into physical hardware, this work establishes a pathway toward physically embedded intelligence for next‐generation electromechanical systems.

## Graphical abstract

Rotational bistability, interfaced through a conductive network with switchable contacts, unifies logic operations and mechanical memory within a rotary electromechanical system. The author demonstrates how serially coupled configurations of rotationally bistable modules enable reprogrammable sequential logic governed by mechanical state transition and electrical connections, a step toward physically embedded intelligence.

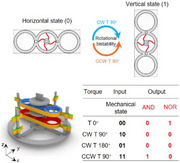

## 1 Introduction

Mechanical computing refers to the unconventional processing of information through mechanically defined states and deformation‐driven transitions [1, 2, 3]. It diverges from conventional systems reliant on rigid components, such as gears or levers, and harnesses intrinsic material deformation to perform logical operations, as demonstrated in material architectures, such as origami and kirigami [4, 5], beam networks [6, 7], rotation‐square mechanisms [8, 9, 10], and soft pneumatic valves [11, 12]. These systems implement digital logic operations, which fall into two categories: combinational logic and sequential logic, a distinction denoting the absence or presence of memory, respectively. The former produces outputs solely from current inputs, independent of any prior state [4, 8, 13, 14], whereas the latter exhibits memory, where outputs depend on both the current inputs and the history of prior states [7, 15, 16]. Sequential logic underpins common functional blocks like counters, registers, and finite state machines (FSMs) that consist of flip‐flops and logic gates, defining transition rules between discrete states [17].

Recent progress in mechanical computing has enabled the development of bistable mechanisms that function as non‐volatile memory [9, 18, 19, 20] for sequential logic [21, 22]. Each bistable mechanism possesses two stable states, which can represent bits (0 and 1) for information storage. When serially coupled, a multistable architecture with stiffness gradients can exhibit deterministic deformation sequences with tunable memory behaviors [23, 24]. Moreover, with an appropriate design of the bistable bits and their interactions, they can perform programmable operations that depend on memory [25, 26]. The outcome is the attainment of traits of intelligence in a system that can sense, store, and process stimuli, a significant step toward self‐adaptive behavior [15] and self‐learning perception [25].

Mechanical logic systems have made encouraging progress, yet key limitations still impede their ability to deliver more advanced intelligent operations, particularly those requiring sequential decision making. First, realizing such functionality requires a system to simultaneously store internal states and process information in a temporally ordered manner in response to external stimuli. Current mechanical computing architectures, however, typically face a fundamental trade‐off: systems designed to perform logic operations often lack intrinsic memory and sequential state evolution [8, 13, 27, 28], whereas mechanically memory‐enabled systems generally support only limited computational complexity [21, 22, 23, 26, 29]. This disconnect highlights the need for mechanical architectures that can integrate state storage with sequential information processing. Second, most existing mechanical logic systems rely on linear actuation, which is fundamentally limited by finite stroke lengths and constrained geometric layouts [15, 22, 23]. These limitations hinder scalability and cap the achievable complexity of sequential operations. In contrast, rotary bistable mechanisms [30, 31, 32] offer a compelling alternative: they enable bidirectional (clockwise/counterclockwise) control and provide a far broader actuation space. Rotation also improves system sensitivity and responsiveness to angular inputs. Yet despite these advantages, angular displacement remains largely unexplored as a primary computational input in mechanical computing architectures. Third, the computational functionality of most mechanical logic systems is fixed at the time of fabrication, severely limiting their adaptability to change on the fly their functionality and properties for varying operating conditions [21, 23, 25, 26]. As a result, enabling reprogrammable sequential decision‐making—where both state evolution and transition pathways can be modified post‐fabrication—remains a central and largely unresolved challenge in mechanical computing.

Here, we present a rotary electromechanical system capable of performing sequential decision‐making through the integration of non‐reciprocal mechanical memory and combinational electrical logic. The architecture comprises serially stacked bistable modules, whose switchable geometric orientations—corresponding to horizontal and vertical states—encode digital bits (Section 2). These mechanically defined states are interfaced with an electrical network to implement basic logic operations concurrently, including NOT and Buffer logic gates (Section 3). Under an applied torque, the stacked system exhibits sequential bistable snap‐through instabilities, giving rise to non‐reciprocal state transitions that are governed by the stiffness gradient and stacking order (Section 4). The integration between the mechanical state evolution and electrical network further enables sequential decision‐making behavior within a unified electromechanical framework (Section 5). When scaled to a rotary four‐bit finite‐state machine (FSM), the system demonstrates advanced computing functionalities, including digital combination locking, in‐memory electromechanical computing, and reprogrammable control for autonomous vehicle steering (Sections 6 and 7). Beyond these demonstrations, the ability to physically embed non‐reciprocal state evolution and logic within a compact mechanical architecture opens new opportunities for intelligent electromechanical systems operating in environments where conventional electronics face intrinsic limitations. This work demonstrates that mechanically encoded computation offers a viable route to embedding intelligence for  next‐generation electromechanical systems.

## 2 Rotary Bistable Module With Switchable Polarization States

**FIGURE 1:**
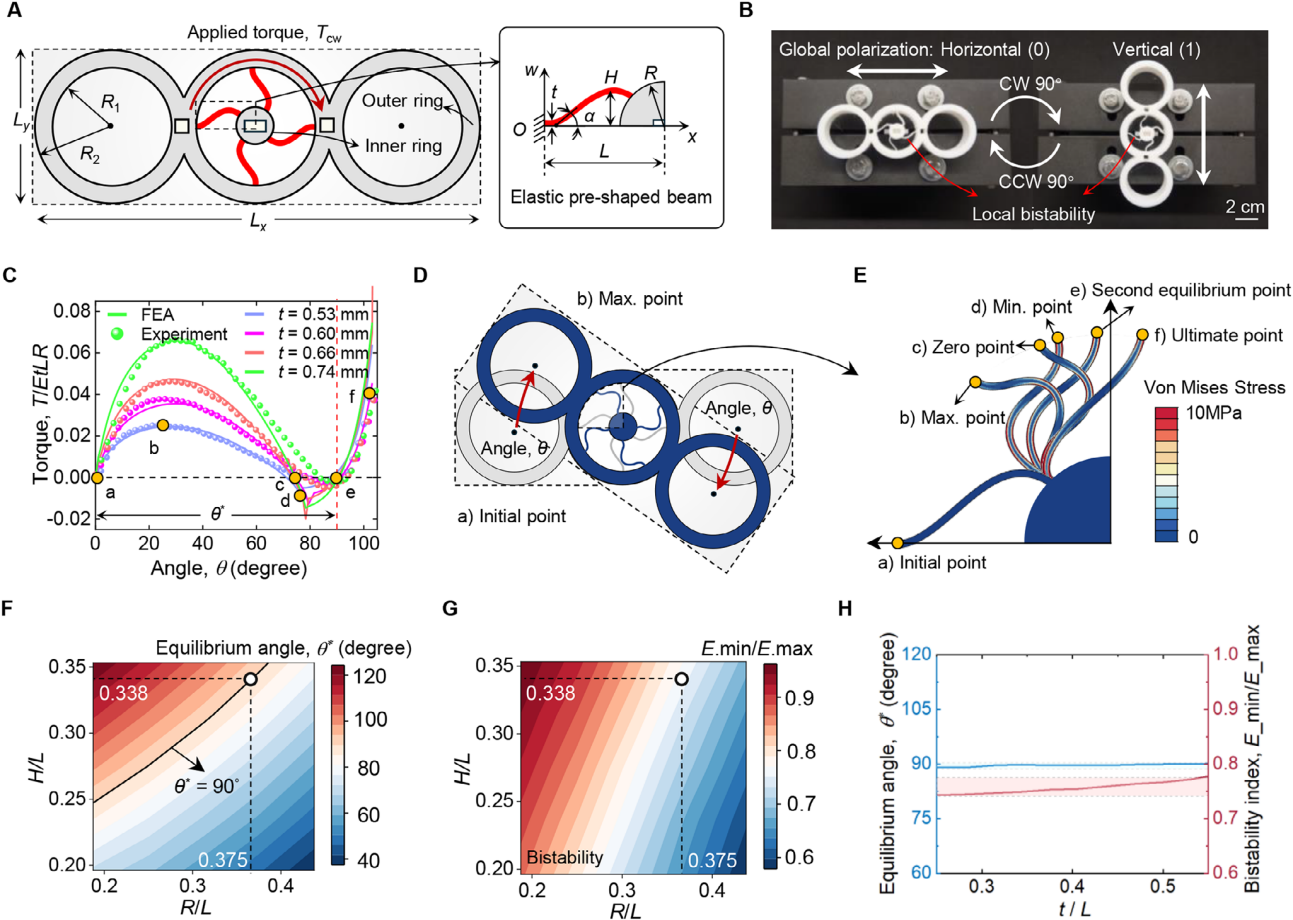
Characterization of the rotary bistable module with switchable global polarization states. (A) Design of the rotary bistable module. (B) Experimental demonstration of the 90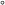 rotation‐driven module. (C) FEA and experimental mechanical characterization of distinct modules with varying in‐plane thickness. (D) Deformation mode comparison between the initial point and the maximum torque point of the pre‐shaped beam. (E) Von Mises stress distribution of six representative deformation modes of the pre‐shaped beam under an applied torque. (F) Contour plot of equilibrium angle, $\theta^{*}$, in the design space defined by non‐dimensional geometry parameters, H/L and R/L. (G) Contour plot of bistability index, $E_{min}/E_{max}$, in the design space defined by non‐dimensional geometry parameters, H/L and R/L. (H) Thickness‐independent equilibrium angle and bistable index properties of the module.

First, we introduce a rotary bistable module, with one degree of freedom, consisting of three contiguous outer rigid rings and one inner rigid ring (Figure 1A). The former is connected to the latter through four pre‐shaped compliant beams (red) with thickness, t that exhibit fourfold rotational symmetry. One end of each beam is connected to the interior circumference of the outer rigid ring, while the other is fixed to the inner rigid ring with radius R. The pre‐shaped beam is characterized by a shape function with height, H, thickness, t, and length, L, as shown in the inset of Figure 1A. The shape of the slender beam governs the bistable rotation of the module, and its non‐dimensional function is described by the buckling mode of a straight beam with clamped‐simply‐supported boundary conditions under axial compression, as illustrated in Figure S1.

To initiate the beam deformation, a torque is applied to the outer rings in the clockwise (CW) direction, $T_{cw}$, while the inner ring remains fixed. The rotation of the outer rings describes the global rotation of the module, while the module undergoes local bistability arising from the geometry incompatibility exercised by the fourfold rotational symmetry arrangement, as shown in Figure 1B. During a rotation of approximately 90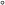, the global orientation of the module transitions from a horizontal (initial) state to a vertical (second) state, defining global polarization states of binary values, 0 and 1, respectively. The switch between them can be manually obtained by applying a 90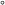 clockwise (CW) or counterclockwise (CCW) rotation to the outer rings.

To characterize the local bistability of the module, Finite Element (FE) simulations and torsional experiments were conducted on four specimens of varying in‐plane thickness, t, from 0.53 to 0.74 mm, for prescribed values of L = 16 mm, R = 6 mm, and H = 5.4 mm. All specimens were placed in their horizontal (0) state with a fixed inner ring, and a rotation angle, θ, was applied to their outer rings. Figure 1C illustrates the evolution of the torque, T, as a function of angle, θ, for the four specimens of varying thickness. First, under CW torque, a pronounced snap‐through bistability is observed, followed by a sharp increase in torque, T, as the modules transition to their second equilibrium state at approximately 90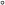 of rotation. Second, the in‐plane thickness, t, of the modules significantly influences the torsional stiffness and the snap‐through threshold, enabling the deterministic control of the deformation sequences when multiple modules with varying in‐plane thickness are stacked in series [24].

By comparing experimental and computational results, we observe that the torque values below zero deviate, a phenomenon primarily due to the rapid curvature reversal of the pre‐shaped beams caused by the elastic constitutive law used in our numerical model, which does not account for viscoelastic effects. In contrast, the viscoelasticity of the base material fabricated using Fusion Deposition Modeling (FDM) mitigates this phenomenon and emerges in the experimental results. Despite this limitation, the elastic constitutive model adopted in our FE simulations successfully captures the salient experimental characteristics of the rotary bistable module (Figure 1C).

To further characterize the deformation modes of the bistable module under an applied torque, T, we select six representative points (denoted as a to f with yellow circles in Figure 1C) pertaining to a beam with a prescribed thickness of 0.55 mm, and study the mode transition of the beams between the initial point and the maximum torque point in Figure 1D. The buckled modes of one beam are illustrated in Figure 1E, which shows the corresponding six deformation modes and von Mises stress distribution. Notably, the curvature of the beam undergoes a reversal between the zero point, c, and the minimum torque point, d, causing a sharp variation in torque value after passing the zero point, as illustrated in Figure 1C.

The equilibrium angle, $\theta^{*}$, labeled in Figure 1C, is a governing parameter that defines the angle span between the initial point and the second equilibrium point. Its value of 90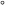 has been intentionally tailored by design through a parametric exploration involving the rationale selection of the dimensionless radius, R/L, and the dimensionless height, H/L, as shown in Figure 1F. The black curve defines the values of the geometric parameters that satisfy $\theta^{*}$ = 90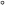. Among its points, we select a demonstrative pair of values, R/L = 0.375 and H/L = 0.338, for each module, as labeled in Figure 1F. In parallel, we generate the counterpart bistability map (Figure 1G) where the bistability index, $E_{min}/E_{max}$, is plotted against the geometric parameters, R/L and H/L. Figure 1F,G illustrate the geometric tunability of the module in attaining a large spectrum of equilibrium angles, $\theta^{*}$, spanning from an acute angle (40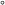) to an obtuse angle (120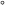), and bistability index. In addition, Figure 1H shows their minimal sensitivity to thickness, t, variations, demonstrating almost complete decoupling between thickness and equilibrium angle. This property is essential for creating serially stacked systems with a thickness gradient, as it allows for the independent tuning of mechanical and geometric properties to achieve complex, tailored deformation sequences. When such deformation sequences are encoded for information storage and processing, they provide robust and finely control over state transitions under angular stimuli, as described later in Section 4.

## 3 A Bistable System With Concurrent Buffer‐NOT Gates and Memory Storage

**FIGURE 2:**
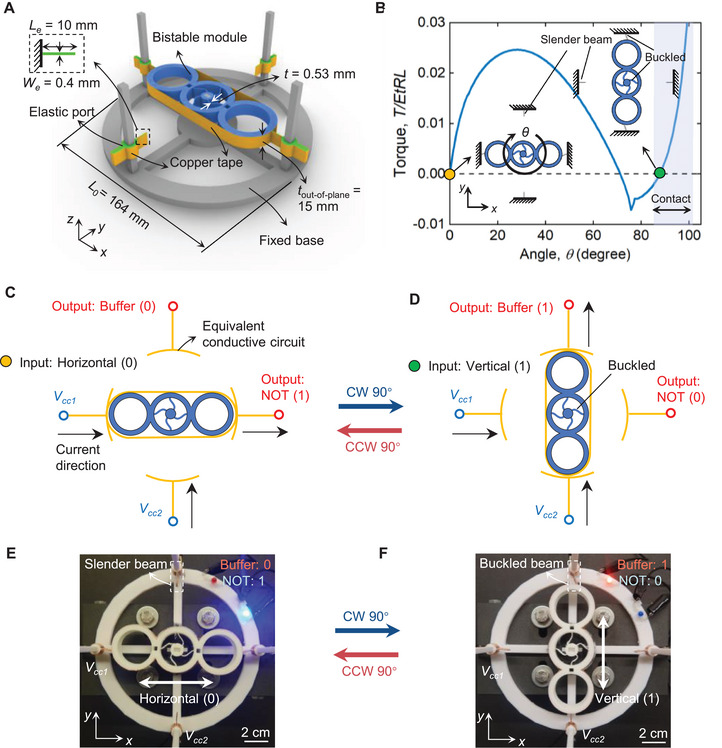
A bistable system with concurrent Buffer‐NOT logic and mechanical memory. (A) Design of a rotary bistable electromechanical architecture. (B) The simulation response of the system when subjected to the torque. (C) Schematic circuitry diagram of the system with input (0) and outputs (NOT: 1 and Buffer: 0). (D) Schematic circuitry diagram of the system with input (1) and outputs (NOT: 0 and Buffer: 1). (E) Experimental photograph of the system in its initial state. (F) Experimental photograph of the system in its second stable state.

We now use the module, set to an equilibrium angle of 90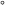, to form a bistable electromechanical system capable of storing one bit of mechanical memory. The bistable system (Figure 2A) consists of the module (in blue), conductive copper tapes (in orange), two pairs of elastic ports (in green), and a rigid base (in grey). The two pairs of elastic ports are positioned at an isometric distance (*x* and *y*) from the center of the base. The extended portion of each elastic port is assumed to act as a straight cantilever beam. Unlike the conductive pathway resulting from the self‐contact properties of rotation‐square mechanisms [8, 13], our copper‐coated system enables the reconfiguration of electrical pathways through switchable contact between the beams and the bistable module. Figure 2B illustrates the torque‐angle response during the system's rotation actuation. In the initial state (yellow point in Figure 2B), the module contacts the beams aligned along the *x*‐direction, causing them to buckle. Upon actuation by a CW 90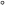, the module snaps through to its second stable state, where it engages beams oriented along the *y*‐direction. The beam geometry is designed to maintain contact with the module in this state, with a contact range from 84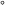 to 102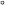, as indicated by the shallow region in Figure 2B. A detailed analysis of the contact process is presented in Figure S4.

Figure 2C,D illustrate an equivalent circuit diagram of the system. The conductive ports enable external electrical connections to the voltage sources ($V_{cc1}$ and $V_{cc2}$ in blue) and the output signals ($Q_{N}$ and $Q_{B}$ in red), while the conductive module functions as a bistable switch, allowing for the connection of one voltage source to one output signal in a given direction. In Figure 2C, the horizontal state of the module (input: 0) establishes the electrical connection along *x*‐direction while remaining disconnected along the ***y***‐direction, yielding outputs of 1 and 0, respectively. In Figure 2D, a CW 90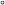 rotation drives the module into the vertical state (input: 1), providing the outputs 0 and 1 along the *x*‐ and *y*‐direction, respectively. Figure 2E,F show the corresponding experimental demonstration. Two LED lights in blue and red signal the on/off status of the Buffer‐NOT logic, respectively. The bistable system described above is capable of performing concurrent Buffer‐NOT gates with a single mechanical input, which executes a NOT operation in the *x* direction and a Buffer operation in the *y* direction. The insets of Figure 2E,F highlight the non‐contact and contact status of the beams located in the *y* direction, respectively.

The rotary bistable mechanism and switchable electrical contact are instrumental in delivering logic operations with non‐volatile memory. The polarization states of the module serve as one‐bit mechanical memory, which acts as input to the electrical logic gates. State transition is achieved by applying a CW/CCW 90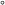 rotation. The system can be stacked in series to form a multistable architecture, enabling sequential information processing as shown in the next section.

## 4 Serially Stacked Electromechanical System

### 4.1 Mechanical Characterization

**FIGURE 3:**
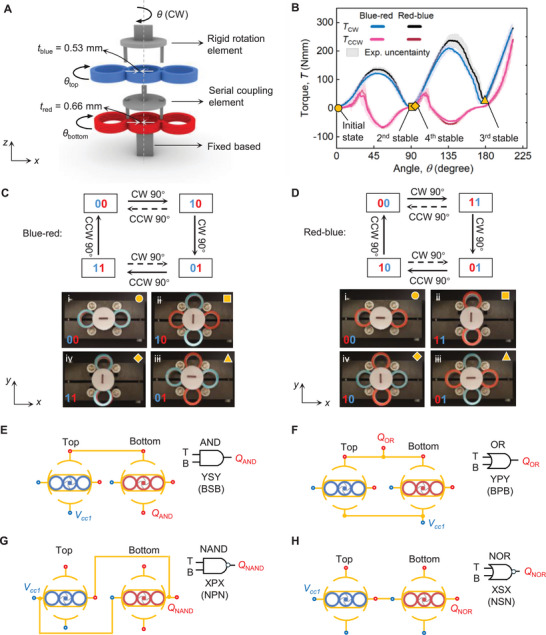
Serially coupled multistable system. (A) Design of multistable mechanical assembly. (B) Mechanical characterization of mechanical assembly with deterministic deformation sequence in experimental measurement. (C−D) Reachable transition pathways and experimental demonstration of the state sequences under different stacking orders. In (C), the modules are stacked in a sequence from top to bottom with increasing in‐plane thickness, whereas in (D), the assembly order is reversed. (E) Electrical connections for AND, OR, NAND, and NOR gates.

We connect two bistable modules in series using a serial coupling element, with each module having a variable in‐plane thickness as illustrated in Figure 3A. The stacking mechanism of two modules is established by rigidly connecting the inner ring of the top module (the blue one with $t_{blue}=0.53mm$) to the outer ring of the bottom module (the red one with $t_{red}=0.66mm$) through a serial coupling element (grey). A rigid rotating element (grey) with the input rotation angle, θ, is mounted on the outer ring of the top module, and the inner ring of the bottom module is fixed to the base.

Figure 3B illustrates the experimental evolution of the torque, T, as a function of the rotation angle, θ, for the mechanical assembly with two dissimilar stacking orders (blue‐red and red‐blue). The assembly delivers sequential bistable snap‐through instabilities with each state transition requiring a 90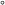 rotation. Due to the thickness variation between the blue and red modules, the system exhibits a deterministic deformation sequence with four stable states [24]. Under CW rotation, the blue module undergoes local buckling first, followed by the red module. On the other hand, during CWW rotation, the buckled beam (blue) snaps back to its initial state first, followed by the red one. There is a slight difference at the peak between the two curves, caused by the torque transmission within the assembly under the two stacking orders.

Figures 3C,D present two groups of polarization state transitions, as captured from the experimental snapshots. Two digital bits are used to represent the polarization states, with each bit referred to by the color of the respective module. Figure 3C illustrates the binary transition sequence of the mechanical assembly, stacked in order of increasing in‐plane thickness from top to bottom: blue to red. The solid arrow indicates the transition sequences: [00‐10‐01‐11‐00] shown in Figure 3C.i–iv. The dashed arrows represent alternative transition pathways. Due to the serial coupling mechanism, the rotation of the lower‐layer module can drive changes in the polarization state of the upper‐layer module, enabling reprogrammable state transitions within the assembly, as opposed to a deformation sequence that is thickness dependent and can only be programmed prior to fabrication [33]. In Figure 3D.i–iv, the reversed stacking sequence configuration (red‐blue) produces the opposite transition sequence: [00‐11‐01‐10‐00], a result demonstrating that the mechanical assembly delivers a static, non‐reciprocal transition sequence that breaks spatial inversion symmetry [34, 35, 36].

The above results demonstrate that our serially stacked mechanical assembly exhibits an in situ switchable transition sequence with static non‐reciprocity, governed by both deterministic deformation sequences and stacking order. The non‐reciprocity enables the emergence of complex state transition patterns with relatively few components. The reprogrammable sequential logic framework provides a powerful tool to effectively characterize the sequential response of the multistable system, capturing complex interactions endowed with memory. Video, S1, demonstrates the relationship of torque‐angle of the assembly with two stacking orders, as well as the binary transition sequence.

### 4.2 Electrical Connection

The two‐layer system incorporates two mechanical inputs (top and bottom) and two pairs of Buffer‐NOT logic elements. It can perform digital logic operations, including AND, OR, NAND, and NOR, by electrically connecting contact ports in series or parallel [8, 13]. To simplify the notation, we denote here the NOT and Buffer logic elements as X and Y gates, respectively, corresponding to their construction along the *x*‐ and *y*‐directions. Table 1 shows the electrical connections to obtain the fundamental digital logic gates. As shown in Figure 3E,F, two Y (Buffer) gates connected in series form an AND (YSY) gate, whereas two Y (Buffer) gates arranged in parallel generate the OR (YPY) gate. Similarly, two X (NOT) gates are used to construct NAND (XPX), and NOR (XSX) gates in parallel and serial connections, respectively, as illustrated in Figure 3G,H. Figure S6 reports the digital logic gates for all outputs. The result demonstrates that the electrical connection of the system supports all basic logic gate operations when connected in an electrical logic network, enabling complex digital functions.

**TABLE 1:** Design principle of digital logic gates.

	**X (NOT**)	**Y (Buffer)**
**S (Series)**	**XSX (NOR)**	**YSY (AND)**
**P (Parallel)**	**XPX (NAND)**	**YPY (OR)**

## 5 Two‐State Electromechanical Device With Programmable Step Control

**FIGURE 4:**
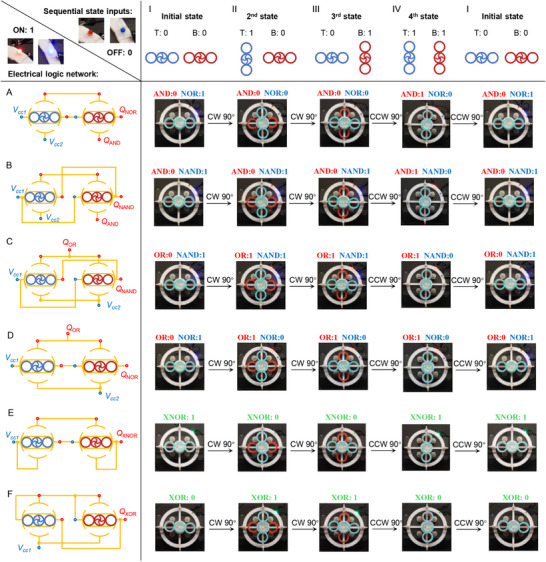
Characterization of the seamless integration of digital logic gates and memory within a multistable system. (A−F) Electrical logic network for AND‐NOR, AND‐NAND, OR‐NAND, OR‐NOR, XNOR, and XOR gates. (I) Initial configuration of the system with the mechanical input of 00. (II) Second stable configuration of the system with the mechanical input of 10. (III) Third stable configuration of the system with the mechanical input of 01. (IV) Fourth stable configuration of the system with the mechanical input of 01.

Building on the non‐reciprocal transition sequences and digital logic gates presented in Section 4, we now integrate these functionalities into a two‐layer multistable system with a programmed stacking order, enabling sequential decision‐making behavior. During continuous rotation, the two‐bit mechanical states are fed into the electrical network, producing two‐bit electrical outputs. The resulting transition sequences exhibit history‐dependent properties that embody the core functionality of sequential logic. To visualize real‐time electrical output, two LED lights—red and blue—are used: a dark LED represents a logical value of zero, whereas an illuminated LED stands for a logical value of one, as shown in the left corner of Figure 4. In this table (Figure 4), the input parameters, i.e., mechanical transition sequence and electrical connections, are arranged along the first row and first column, respectively. The tabulated values represent the system outputs (two‐bit electrical state) for each corresponding input pair. The first row specifies the mechanical state inputs ([00‐10‐01‐11‐00]) generated by a multistable assembly with a stacking order from top to bottom in order of increasing thickness (Figure 3C). The first column lists the electrical inputs derived from the logic network, which employs two pairs of Buffer‐NOT gates to realize AND‐NOR, AND‐NAND, OR‐NAND, OR‐NOR, XNOR, and XOR gates, respectively (Figure 4A–F).

From the construction principle of Table 1, we connect two Y and X gates in series to produce the AND‐NOR gates in Figure 4A. Cell(A, I) displays the outputs (01) of the concurrent AND‐NOR gate with an initial mechanical input of 00, as shown in the experimental snapshot where the red LED is “off” and the blue LED is “on”. Upon a CW 90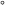 rotation, the system reaches its second state, with a mechanical input of 10 and an electrical output of 00, as calculated by AND‐NOR gate. (Cell A, II). With rotation, the system sequentially executes AND‐NOT logic operations, generating the following electrical transition sequence: [01‐00‐00‐10‐01] from cell (A, I) through cell (A, IV) and back to cell (A, I). Other logic gates, including AND‐NAND, OR‐NAND, and OR‐NOR, are constructed by electrically connecting two pairs of X and Y gates, following the design principles outlined in Table 1. These gates are illustrated in the first row of Figure 4B–D. Specifically, the XNOR gate is realized by first connecting two Y and X gates in series to form AND (YSY) and NOR (XSX) gates, which are then arranged in parallel to yield the XNOR logic output (Figure 4E). Similarly, the XOR gate is constructed by initially connecting two Y and X gates in parallel, followed by the series connection of the resulting OR (YPY) and NAND (XPX) gates to generate the XOR logic output (Figure 4F). The corresponding mechanical configurations and sequential electrical state transitions are summarized in the tabulated values under their respective computations.

The combined mechanical state sequence and electrical logic network reproduce the truth‐table behavior of ideal logic gates. Hence, our multistable architecture delivers programmable sequential logic under rotational actuation, coupling non‐reciprocal mechanical memory with electrical pathways to process sequential inputs. Videos, S2–S4 demonstrate the system's programmable sequential decision‐making functions with the AND‐NOR, OR‐NAND, and XNOR logic gates, respectively.

## 6 Rotary Four‐Bit FSM With Reprogrammable Electromechanical Computations

**FIGURE 5:**
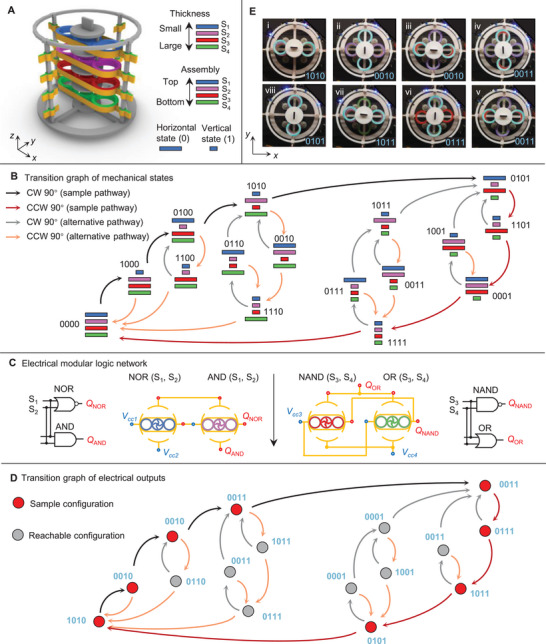
Rotary four‐bit FSMs with complex transition sequences through electromechanical computations. (A) Configuration of a four‐bit FSM. The four bistable modules are assembled in a sequence following an increasing in‐plane thickness. (B) Transition graph of mechanical states. (C) Electrical modular logic network with computations of AND $\left(S_{1},S_{2}\right)$, NAND $\left(S_{1},S_{2}\right)$, OR $\left(S_{3},S_{4}\right)$, and NOR $\left(S_{3},S_{4}\right)$. (D) Transition graph of electrical outputs. (E) Experimental snapshots with multistable configurations and electrical outputs, where four blue LEDs indicate the output states.

Finite state machines (FSMs) are defined by the transition rules that govern the discrete states, each with functional outputs [16, 21]. The serially coupled system offers a robust and scalable framework for multi‐bit information processing, which can be described as an FSM with multiple logic outputs. Figure 5A shows a four‐bit FSM with four modules, stacked from top to bottom in order of increasing thickness. Based on single module experiments (Figure 1C), the modules are expected to snap‐through and snap‐back sequentially in the same order: blue‐purple‐red‐green. Under rotational actuation, the four‐bit FSM can simultaneously execute four distinct types of logic operations, where the rotational protocols and electromechanical configuration determine the resulting output sequence.

Figure 5B shows the transition graph of the mechanical states of the FSM, illustrating all $2^{4}=16$ stable configurations. The stacking order and the deterministic deformation sequence of the modules govern the transition rules. For example, in the transition from 1000 to 0100, the second CW 90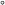 rotation triggers the deformation of the purple module, while it alters the polarization state of the upper one, the blue module. The logic output of the FSM: AND $\left(S_{1},S_{2}\right)$, NAND $\left(S_{1},S_{2}\right)$, OR $\left(S_{3},S_{4}\right)$, and NOR $\left(S_{3},S_{4}\right)$, is dictated by the electrical network, shown in Figure 5C. In these logic gates, the first two mechanical states $\left(S_{1},S_{2}\right)$ are inputs to NOR and AND gates, while the last two states $\left(S_{3},S_{4}\right)$ are inputs to NAND and OR gates. The four‐bit mechanical states transition graph provides four input arguments to the logic gates to create the four‐bit electrical outputs, as illustrated in Figure 5D. The black and red arrows in Figure 5B,D represent the loading and unloading pathways observed in the experiments, as shown in Figure 5E, while the grey and orange arrows indicate alternative possible transition pathways. Figure 5E shows the corresponding experimental demonstration, where four blue LED lights indicate the outputs. The sequence of mechanical rotations required to reach a specific sequence can be modified by altering the electromechanical configuration. Figure S8 illustrates reprogrammable transition rules under dissimilar configurations.

The operating mechanism of the system enables mechanical and electrical logic, which produces complex transition sequences. This allows the realization of a digital combination lock, where the FSM only transitions to the desired ‘unlock’ state after a precise sequence of electromechanical inputs [22]. The electromechanical operation further enhances its resistance to tampering, as the complexity of the transition sequence makes it significantly more difficult to guess or brute‐guess without explicit knowledge of the correct configuration pathway. This opens opportunities for mechanical pattern recognition, secure access control in smart materials, and physically encoded security. While the above is promising, we also note that using higher bit counts (six or more) in our stacked FSMs creates inherent challenges due to the limited feasible range of thickness in the pre‐shaped beams. Beam thicknesses must both fall within a range that ensures robust bistability, and have a sufficiently large stiffness gradient across stacked bits to transition between states correctly.

### 6.1 Applications 1: Reprogrammable FSM with Sequential Half‐Adder Computing

**FIGURE 6:**
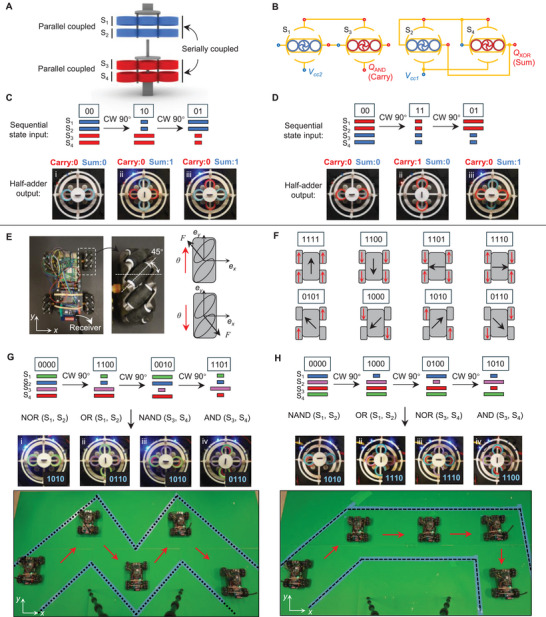
(A−D) Reprogrammable FSMs with sequential half‐adder computing: continuous execution of half‐adder computing with memory storage. (A) Mechanical assembly. (B) Electrical logic network for carry and sum bit output. (C−D) Experimental demonstrations of the successive states with half‐adder electrical outputs under the different stacking orders. (E−H) Reprogrammable digital logic control of a Mecanum‐wheeled car. (E) Mecanum wheels composed of rollers with 45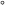 inclination enable omnidirectional movement of the car. (F) The encoding rules for controlling the omnidirectional movement of the car. (G−H) Experimental demonstration of the reprogrammable trajectory control of the car.

To demonstrate advanced electromechanical computing, we implement the design principle into an FSM capable of sensing rotational stimuli, continuously performing high‐level arithmetic operations (half‐adder), and memorizing the current state. In Figure 6A–D, we present a mechanical state machine whose inputs are sent to a half‐adder to produce a sum of the state bits. In this application, the computing unit captures mechanical input and then electrically outputs the corresponding sum and carry bits for each state.

Figure 6A shows the assembled mechanism where the modules with identical in‐plane thickness (indicated with identical color) are connected in parallel, and the top two and bottom two modules are then connected in series. The parallel connection enables synchronous rotation of the paired modules, allowing the four‐bit system to be represented using two digital bits. The electrical logic network for a half‐adder is shown in Figure 6B, where $S_{1}$ and $S_{3}$ are computed by the AND gate to generate the carry output, while $S_{2}$ and $S_{4}$ are processed by the XNOR gate to produce the sum output. The experimental results demonstrate the computing process with reprogrammability, from the mechanical sequence of [00‐10‐01] to the electrical addition sequence of [00‐01‐01] (Figure 6C), and from [00‐11‐01] to [00‐10‐01] (Figure 6D).

The reprogrammable system presented here performs complex digital computing, with potential applications in in‐memory electromechanical computing, where information storage and computing are seamlessly integrated within the system [25, 37]. It supports multiple‐bit information storage and continuous execution of higher‐level arithmetic functions, including multi‐bit addition, subtraction, and multiplication [13], enabling the design of intelligent electromechanical devices, such as MEMS.

### 6.2 Application 2: Reprogrammable Digital Control of a Mecanum‐Wheeled Car

Controlling the transition pathways between states, while generating electrical outputs, enables applications in digital device control. To demonstrate this functionality, we integrate a four‐bit computing unit with a Mecanum‐wheeled car to demonstrate its digital control functions, as shown in Figure 6E–H. The Mecanum wheels are a specialized type that allows the car to achieve omnidirectional movement by independently controlling the rotation direction of each wheel (Figure 6E). The movement direction of the car originates from the resultant force of four wheels, which can be encoded using four bits, as shown in Figure 6F. The encoding rules of each bit, corresponding to distinct directions of movement, are detailed in Figure S9.

Driven by the rotation angle, four‐bit electrical signals that guide the car along a targeted trajectory are generated by the computing unit, transmitted in real‐time via a wireless transmitter module, and received by the car equipped with a corresponding receiver (Figure 6E). In our experiment, an inverse design is carried out to construct the electromechanical configuration of the unit, enabling on‐target trajectory forming a “M” pattern as illustrated in Figure 6G. Reprogrammability is accomplished by in situ altering the electromechanical configurations to obtain distinct transition sequences, a strategy leveraged to direct the car along the trajectory shown in Figure 6H. Video S5, demonstrates the attainment of the target trajectory through the four‐bit system.

The concept presented here for reprogrammable digital control can transform mechanical stimuli into real‐time digital control in electrical devices that require sequential state transitions. These systems offer a pathway for embedding intelligence into physical systems, where logic and memory are seamlessly integrated, enabling applications that demand real‐time, multi‐channel decision‐making.

## 7 Conclusion

This work presents a rotary multistable electromechanical system capable of performing sequential decision‐making. The core strategy is to use the geometric orientation of the bistable modules as digital bits, interfacing via a conductive network with switchable contacts to unify logic operations with mechanical memory within a single architecture (Figure 4; Figure S6). The serial coupling of modules enables non‐reciprocal state transition, producing history‐dependent sequences dictated by both the deformation pathway and stacking order, thereby enhancing the system's adaptivity for information processing. Furthermore, the system detects rotational inputs, a sensing modality that remains largely unexplored in current mechanical computing [7, 13, 21, 25]. Our rotary computing framework can integrate with rotation‐driven mechanisms [31, 32], such as gear clusters, thereby opening avenues for parallel [38] and distributed electromechanical computing.

The integrated conductive network allows the system to sequentially execute the higher‐level arithmetic with memory storage, such as multi‐bit addition, subtraction, and multiplication [13]. The proposed bistable modules, exhibiting a wide range of equilibrium angles from 45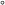 to 120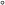, can be strategically utilized to enhance the information processing density. As illustrated in Figure S7, an alternative 45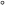 rotary configuration enables a single mechanical input to generate multiple Buffer‐NOT outputs, indicating that the complex combinational logic can be realized with fewer mechanical modules.

The integration of mechanical and electrical computation enables the system to function as a finite‐state machine (FSM), with well‐defined and programmable transition rules [15]. Under prescribed angular displacements (i.e., clockwise or counterclockwise 90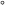 rotations), mechanical bits are sequentially encoded and fed into the electrical logic network, forming an intelligent sequential computing unit that unifies rotation sensing (analog‐to‐digital conversion [39]), sequential information processing, mechanical memory storage, and adaptive digital control. The system's capabilities are experimentally validated and demonstrated for sequence‐sensitive digital combination locks, in‐memory electromechanical computing, and reprogrammable digital control. In summary, this work contributes to physically embodied computation, laying the foundation for intelligent electromechanical devices with advanced and reprogrammable computing. The framework integration supports adaptive control systems—including autonomous robotic platforms—that can dynamically readjust tasks in situ in response to environmental changes, without requiring hardware modification.

## 8 Experimental Section

### 8.1 Sample Fabrication

The bistable modules in Figure 1 and the elastic ports in Figure 2 were fabricated with a QIDI TECH i‐fast (QIDI, Wenzhou, Zhejiang, China) via Fused Deposition Modelling (FDM) out of the white TPU filament. The rigid elements in grey of the four‐bit FSM in Figure 5 were fabricated with an Anycubic Vyper (Anycubic, China) via FDM out of the white PLA filament. The electrical contacts were constructed from double‐sided conductive copper tape, which was fabricated by DAOKI, China. The Mecanum‐wheeled car kit was fabricated by Freenove, China. The embedded wireless sense modulus (EVAL‐433‐KH3) was fabricated by TE Connectivity. More details about the fabrication can be found in Figure S10.

### 8.2 Uniaxial Torsional Test

The quasi‐state torsional tests of the bistable modules and mechanical assemblies in Figures 1 and 3 were performed under an angle‐controlled torque with a rate of 2 deg/s by a stepped motor with integrated driver and controller (23 MDSI, Anaheim Automation, China), and the torque‐angle curve was measured by a rotary torque sensor (ATO, China). For each specimen, we perform the loading test three times and obtain the average response and the experimental uncertainty range. The details about the torsional test and experimental setup could be found in Figure S11.

## Conflicts of Interest

The authors declare no conflicts of interest.

## Supporting information


**Supporting File**: advs73847‐sup‐0001‐SuppMat.docx.

**Supplemental Video 1**: advs73847‐sup‐0002‐VideoS1.mp4.

**Supplemental Video 2**: advs73847‐sup‐0003‐VideoS2.mp4.

**Supplemental Video 3**: advs73847‐sup‐0004‐VideoS3.mp4.

**Supplemental Video 4**: advs73847‐sup‐0005‐VideoS4.mp4.

**Supplemental Video 5**: advs73847‐sup‐0006‐VideoS5.mp4.

## Data Availability

The data that support the findings of this study are available from the corresponding author upon reasonable request.
