# Supplementary material for: Rotary Electromechanical System Integrating Non‐Reciprocal Memory and Combinational Logic
Source: Adv Sci (Weinh). 2026 Jan 22;13(17):e22133. doi: 10.1002/advs.202522133 (PMC13042823; doi:10.1002/advs.202522133)
Supplement: Supplementary file 1 — Supporting File: advs73847‐sup‐0001‐SuppMat.docx. [file ADVS-13-e22133-s002.docx]

SUPPORTING INFORMATION

Rotary electromechanical system integrating non-reciprocal memory and combinational logic

Shujia Chen, Don Straney and Damiano Pasini*

*Department of Mechanical Engineering, McGill University, Montreal, Quebec H3A 0C3, Canada* ∗Corresponding author: Damiano Pasini; [damiano.pasini@mcgill.ca](mailto:damiano.pasini@mcgill.ca)

**I Finite Element Analysis**

**i)** *Torque-angle relation of the rotation-driven bistable module*

The deformable element of our rotational bistable module (**Figure** S1-a) is composed of four pre-shaped beams with 4-fold rotational symmetry, which delivers rotation-driven bistable properties. The governing equation of the pre-shaped beam, originating from the first buckling mode of a straight, slender beam with a thickness, *t*, subjected to axial compression is shown in **Figure** S1-b. The equation describing a straight beam subjected to the compressive load *F* is given by:

$\text{EI}\frac{\text{d}^{\text{4}}\text{w}}{\text{d}\text{x}^{\text{4}}}\text{ }\text{+}\text{ }\text{F}\frac{\text{d}^{\text{2}}\text{w}}{\text{d}\text{x}^{\text{2}}}\text{ }\text{=}\text{ }\text{0}$ (1)

where *w* is the lateral displacement of the midplane of the beam, *E* is the elastic Young’s modulus, and *I* is the moment of inertia of the beam. The clamped-simply-supported boundary conditions are defined as:

$\text{w}\text{(0)}\text{ }\text{=}\text{ }\text{w}^{\text{'}}\text{=}\text{ }\text{0,}\text{ }\text{w}\text{(}\text{L}\text{)}\text{ }\text{=}\text{ }\text{w}^{\text{''}}\text{(}\text{L}\text{)}\text{ }\text{=}\text{ }\text{0}\text{ }$ (2)

The generous solution of equation (1) is:

$\text{w}\text{0}\text{(}\text{x}\text{)}\text{ }\text{=}\text{ }\text{A}\text{cos}\text{kx}\text{ }\text{+}\text{ }\text{B}\text{sin}\text{kx}\text{ }\text{+}\text{ }\text{Cx}\text{ }\text{+}\text{ }\text{D}$ (3)

By substituting equation (2) into equation (3), and choosing the first buckling mode (*k*_1_ = 1.43π/L) for the straight beam, we obtain the shape function of the curved beam as:

$\text{w}\text{0}\text{(}\text{x}\text{)}\text{ }\text{=}\text{ }\text{B}\text{(-}\text{k}\text{1}\text{L}\text{cos(}\text{k}\text{1}\text{x}\text{)}\text{ }\text{+}\text{ }\text{sin(}\text{k}\text{1}\text{x}\text{)}\text{ }\text{+}\text{ }\text{k}\text{1}\text{ (}\text{L}\text{-}\text{x}\text{)}\text{)}$ (4)

where the shape of the curved beam is controlled by two parameters, the coefficient *B* and the beam span *L*. The coefficient *B* exhibits a monotonically increasing relationship with the apex height of the pre-shaped beam, *H* [1, 2]. When constructing the bistable module, a portion of the pre-shaped beam is embedded into the rigid inner ring with a radius, *R*, as shown in **Figure** S1-a. Consequently, the inner ring’s radius, *R*, also plays a crucial role in determining the mechanical properties of the module.


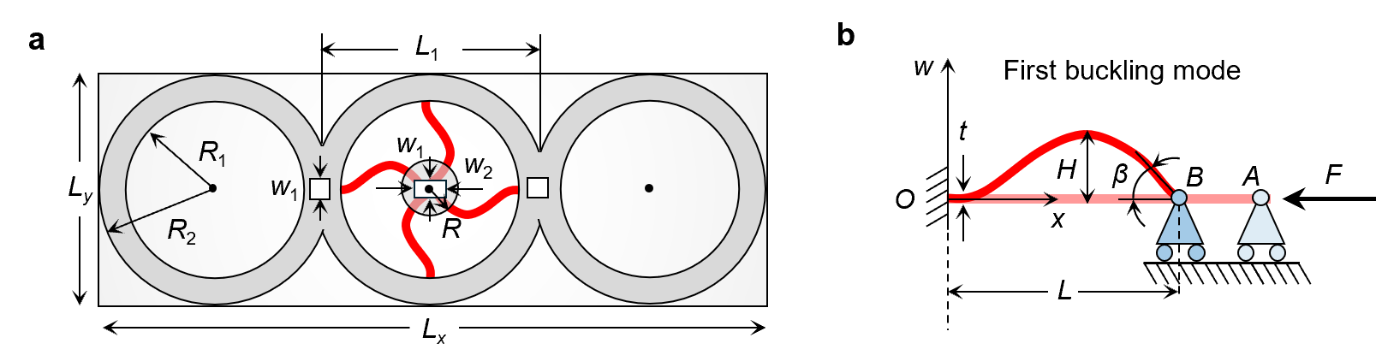


FIG. S1: (a) Illustration of the rotation-driven bistable module comprising four pre-shaped beams with 4-fold rotational symmetry, one inner ring and three connected outer rings, where *L_x_ =* 120mm, *L_y_ =* 42mm, *L*_1_ *=* 40mm, *R*_1_ *=* 16 mm, *R*_1_ *=* 21 mm, *w*_1_=4.2 mm, *w*_2_=6.2 mm, out-of-plane thickness of 15 mm. (b) Configuration of the pre-shaped beam, resulting in the first buckling mode of a straight beam subjected to axial compression, where *t* = 0.53 mm, *L* = 16 mm, *H* =5.4 mm, *R* = 6 mm, *β* = 45°.

To investigate the rotation-driven bistable behaviour and analyze the effect of the geometry parameters on the mechanical properties of our module design, we create a 2D Finite Element (FE) (Abaqus, Dassault Systems) with dimensions shown in **Figure** S1-a, and out-of-plane thickness of 15.0 mm. A linear-elastic material model is employed to describe the constitutive behavior of the base material, Thermoplastic Polyurethane (TPU) [3-5]. See Section **V**, **Material Characterization** for details about the TPU properties. To enhance numerical convergence, we incorporate the Rayleigh-damping with a mass proportional factor of 1 for damping the lower frequencies and a stiffness proportional factor of 0.01 for damping possible higher frequencies. To ensure reliable results for our module which features abrupt curvature variations, we adopt refined mesh with quadratic plane stress triangle elements (CPS6), as shown in **Figure** S2-a. The mesh density is refined in the highly deformable elements in regions with sharp changes in geometry, whereas a coarser mesh is applied to the rigid regions to optimize computational efficiency. A mesh convergence analysis ensures the quality and reliability of the mesh. A general contact condition is assigned to the entire model, with normal behavior defined as hard contact and tangential behaviour govern by a friction coefficient of 1.2, respectively [6]. To enable the rotation of the outer rings with respect to the inner ring, which is set to ground, in the simulation, we couple the former to one reference point through the beam connection within a multi-points constraint (MPC) framework. This enables to prescribe the rotation angle directly to the reference point. Given the quasi-static loading conditions our module operates, a *Quasi-static* application within the *Dynamic Implicit* solver, is used, under an initial time increment of 0.01 s and a minimum time increment of 10^-7^ s. Throughout the simulation, the moment and rotation angle of the reference point are monitored to extract the necessary data for the derivation of the non-dimensional *T* – *θ* relationship shown in **Figure** 1C. **Figure** S2-b shows the stress distribution of the module with the applied rotation angle, *θ*.


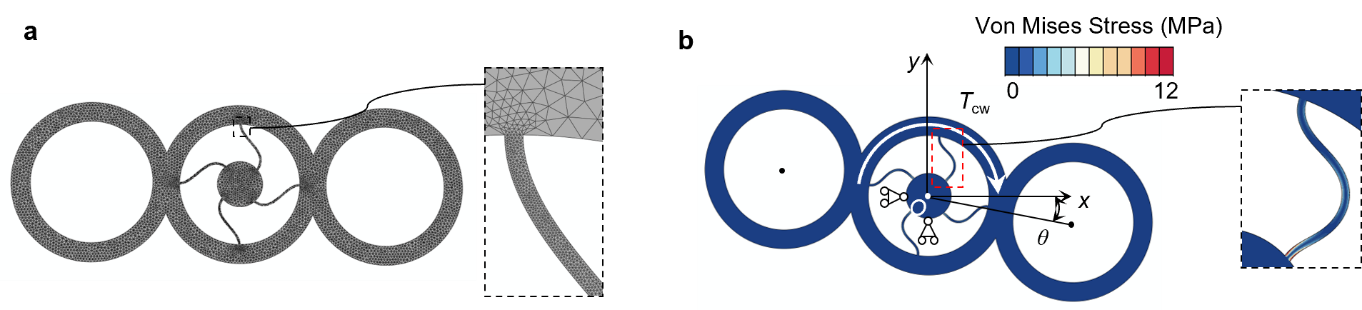


FIG. S2: (a) Mesh of the rotational module (b) Stress distribution with the applied rotation angle, *θ*.

The entire numerical simulation process above is implemented using an in-house Abaqus-python script. The results presented in **Figure** 1C pertains to four module geometries with thickness ranging from 0.53 mm to 0.74 mm. A parametric sweep is then conducted to analyze the effect of two geometry parameters, *H*/*L*, and *R*/*L*, on the equilibrium angle, *θ^*^*, and bistable index, *E*_min_/*E*_max_, as shown in Figures 1F and 1G. Specifically, *H*/*L* varies from 0.18 to 0.44 with increments of 0.26/10, while *R*/*L* from 0.18 to 0.36 with increment of 0.18/10, while the thickness, *t*, is prescribed at 0.53 mm. The equilibrium angle is determined by measuring the span of the angle from point a) to point e) on the non-dimensional *T* – *θ* curve, whereas the bistable index is calculated from the local maximum and minimum points of the strain energy landscape, which is derived through theoretical integration of the non-dimensional *T* – *θ* curve. The contour plot of the equilibrium angle **Figure** 1F exhibits a decreasing trend along the direction when R/H increases. This trend arises from the fact that an increasing R contributes to shortening the deformable portion of the beams, thereby restricting its rotation. On the other hand, a decreasing H reduces the height of the pre-shaped beam, limiting its curvature change. From the parameter study, we select four representative geometries of our rotational module (Figure S3-a) for varying H/L and R/L that achieve equilibrium angle at 45°, 60°, 90°, and 120°. The torque-angle relationship for varying non-dimensional geometry parameters H/L and R/L (Figure S3-a) follows the experimentally observed trend (**Figure** 1F), where a decrease in *R* and an increase in *H* lead to a higher equilibrium angle. **Figure** S3-c show the corresponding deformation modes of each module geometry at its second equilibrium state. Furthermore, we investigate and visualize in **Figure** 1H the role of thickness on both the equilibrium angle and bistable index by varying *t* from 4 mm to 9 mm in increments of 5/10.


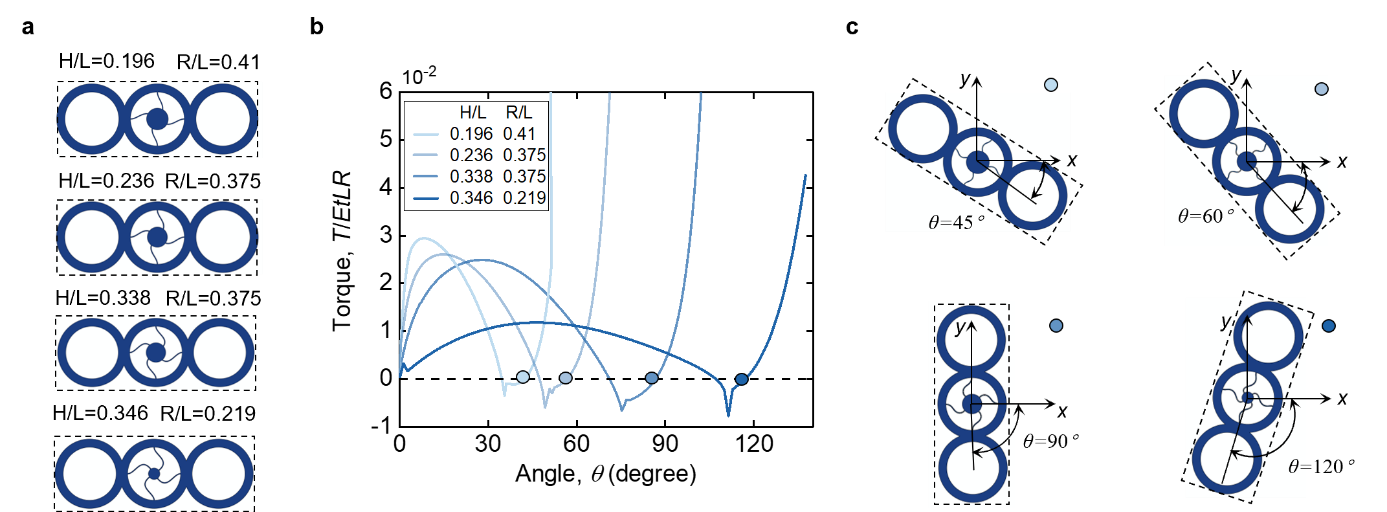


FIG. S3: (a) Four different module designs with varied non-dimensional H/L and R/L. (b) Evolution of non-dimensional torque-angle curves for four module geometries. (c) Deformation modes at the second equilibrium state. Four geometric designs of the module enable equilibrium angle of 45°, 60°, 90°, and 120°, respectively.

**ii)** *Contact analysis between the bistable module and elastic ports*

To analyze the contact behavior between the bistable module and the elastic ports, we model the extended portion of an elastic port as a straight beam with a fixed end (**Figure** 2A). Initially, the module is positioned at an inclined angle, without contact of the elastic ports located along the *x*-direction, as shown in **Figure** S4-a (purple). The simulation process of the contact behavior consists of two steps. In the first loading step (*Dynamic Implicit*, *Quasi-static*), the entire module rotates from the inclined position (**Figure** S4-a) to the horizontal state (**Figure** S4-b), resulting in the buckling of the beams along the *x*-direction, as illustrated in **Figure** S4-b. In the second loading step (*Dynamic Implicit*, *Quasi-static*), a torque (rotation angle-controlled) is applied to the outer ring while the inner ring remains fixed. The module initially loses contact with the elastic ports along the *x*-direction. After undergoing a local bistable snap-through behavior, it establishes contact with the elastic ports along the *y*-direction, reaching its second equilibrium state upon approximately 90° rotation (**Figure** S4-c). The corresponding torque-angle relationship for these two steps is monitored and is diagramed in **Figure** S4-d. The module undergoes two individual contacts with each pair of elastic ports along the *x*- and *y*- directions. The former persists in the angle range 2.1° to 17.3°, while the latter spans 91.4° to 106.6°, as highlighted by the shaded regions. The angles of the three representative configurations shown in **Figure** S4-a are also marked on the torque-angle curve (three colored points) of **Figure** S4-d. **Figure** S4-e is an inset of the curve in Fig. S3-d that shows the start of the contact which causes the original straight cantilever beam to bend upward. Beyond the yellow point, the abrupt change in slope of the torque is mainly caused to the buckling of the pre-shaped beams. The geometry of the straight beam is selected to ensure the occurrence of bistability in the module within the contact region, as shown in **Figure 1G.**


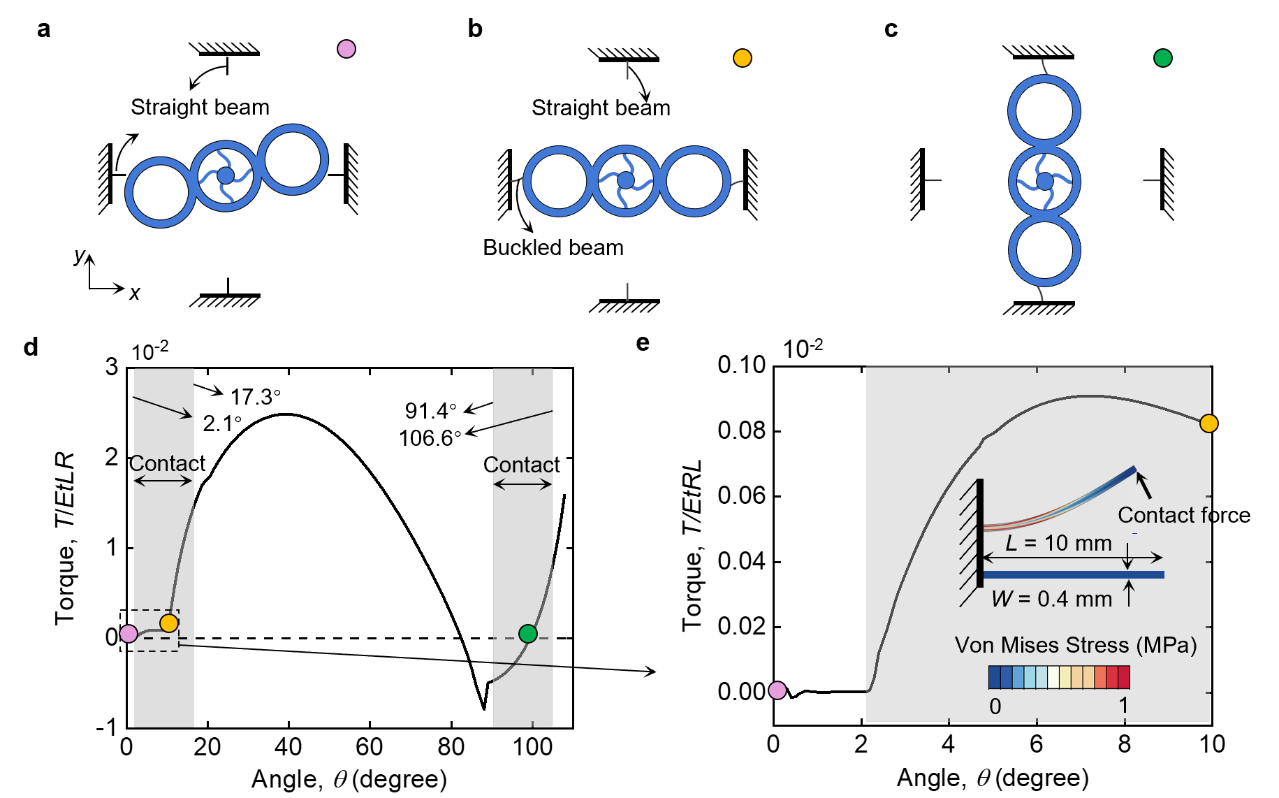


FIG. S4: (a) Initial configuration of the simplified FE model for contact behavior analysis, where although the module is inclined at a certain angle, no contact takes place with the elastic ports yet. (b) Horizontal state of the module, where contact with the elastic ports takes place along the *x*-direction. (c) Vertical state of the module, where contact with the elastic ports takes place along the *y*-direction.

**II Electrical logic network**

**i) Digital logic gates enabled by electrical connections of two building blocks**

Two building blocks provides two sets of NOT-Buffer logic elements & 2-bit binary mechanical memory, which can be used to construct all the fundamental logic gates, including AND, OR, NAND, and NOR, through electrical serial and parallel connections, as illustrated in **Figure** S5. The AND (YSY) and OR (YPY) gates are realized by connecting two Y (Buffer) gates in series and parallel, respectively, as shown in **Figure** S5-a and b. In the case of AND gates in **Figure** S5-a, the circuit outputs 1 only when both modules are in the vertical (1) state; in all other cases, the circuit remains off (0). The NAND (XPX) and NOR (XSX) gates are derived by readily applying De Morgan’s theorem to the AND and OR gate configurations. Specifically, the NAND is the negation of the AND, expressed as the intersection of the two inputs (the top input and bottom input). This is equivalent to the sum of the negation of the top input and bottom inputs. This logical transformation can be physically implemented by parallel connections, as illustrated in **Figure** S5-c. Similarly, by electrically connecting two X (NOT) gates in series, the NOR gate is realized, as depicted in **Figure** S5-d.


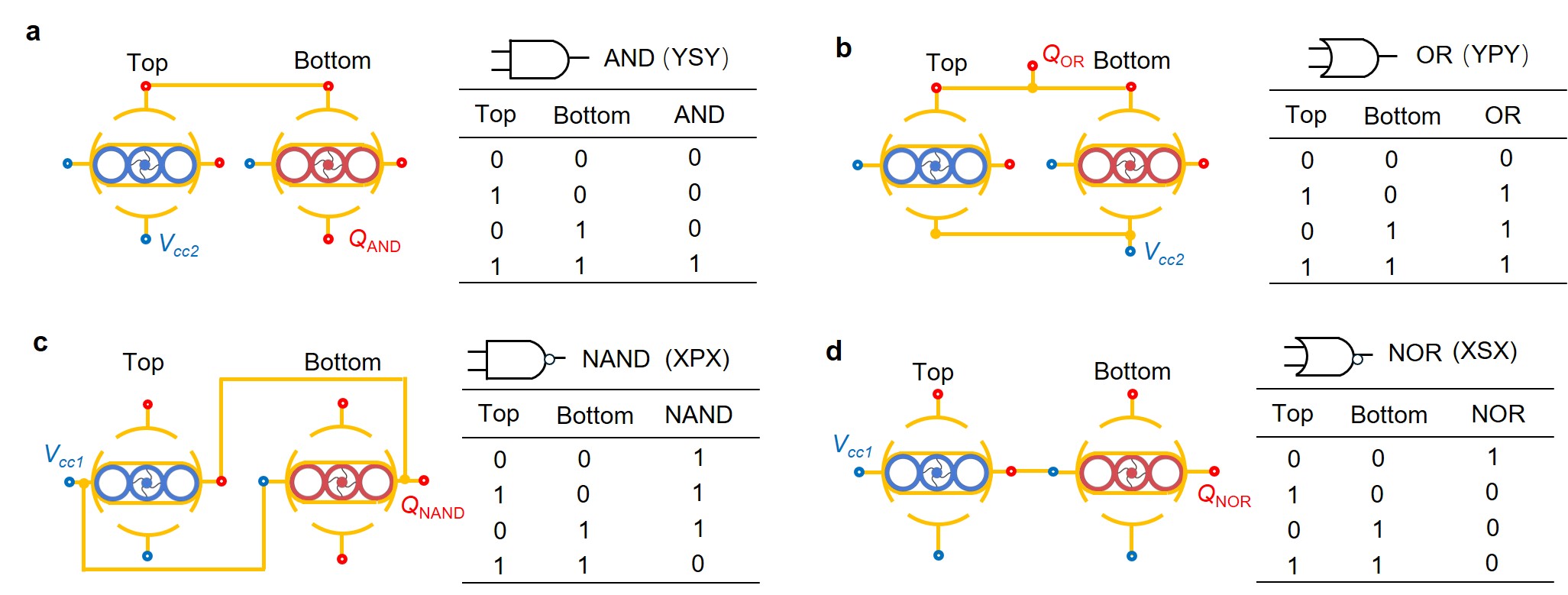


FIG. S5: (a) Two Y gates connected in series to form an AND gate. (b) Two Y gates connected in parallel to form an OR gate. (c) Two X gates connected in parallel to form a NAND gate. (d) Two X gates connected in series to form a OR gate.

**ii) Performing all digital logic operations within a two-layer electromechanical system**


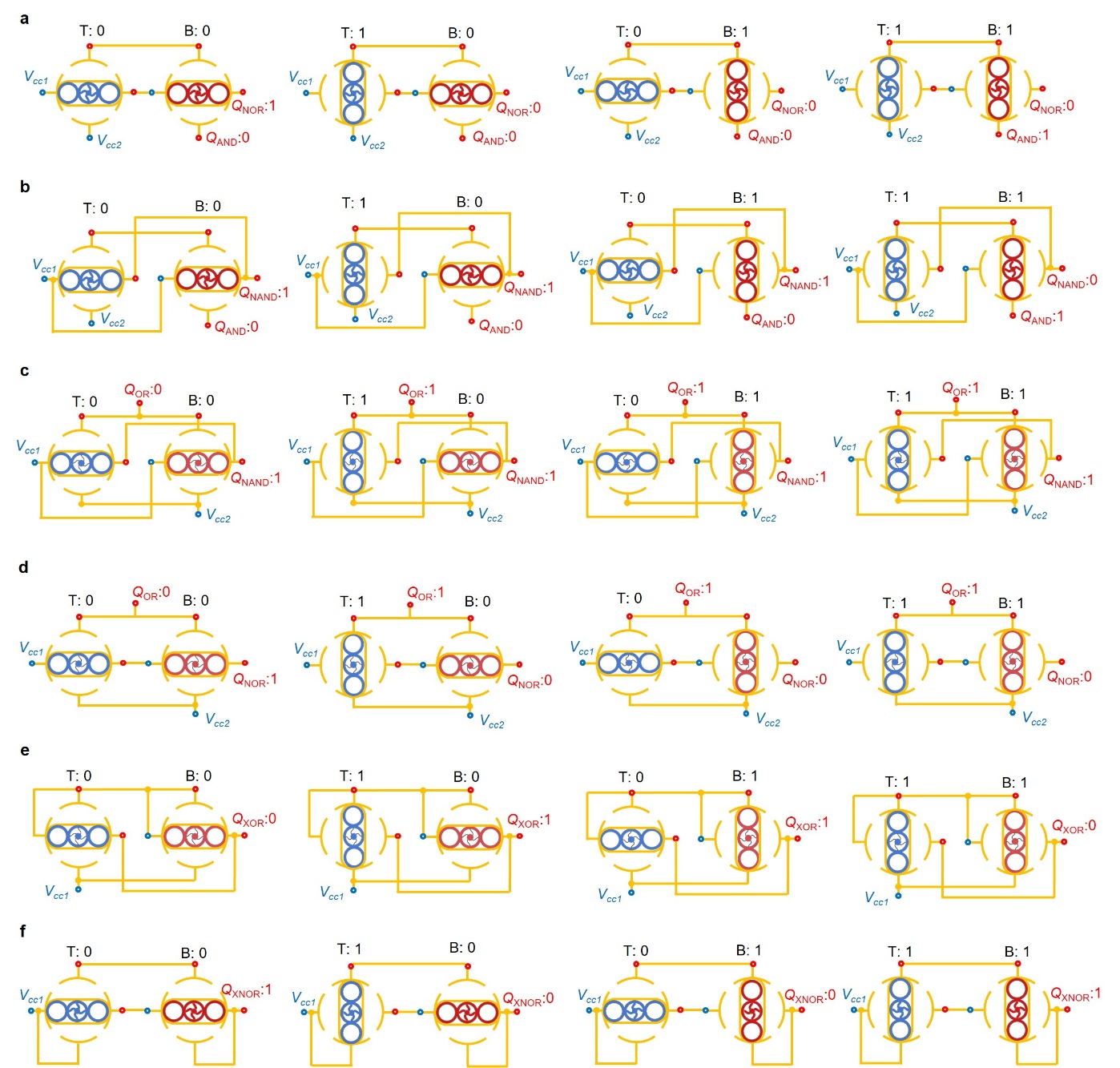


FIG. S6: Output of digital logic gates arising from the system’s four stable states. (a) AND-NOR gate (b) AND-NAND gate. (c) OR-NAND gate. (d) OR-NOR gate. (e) XOR gate. (f) XNOR gate.

**iii)** *45° rotation-driven system with multiple NOT-Buffer outputs*

The electrical logic network is constructed from the NOT-Buffer logic elements, which take their inputs from the 90° rotation-driven bistable module. We next introduce an alternative 45° rotation-driven bistable module, which enables two groups of NOT-Buffer logic elements with a single mechanical input. The module consists of four connected outer rings, one inner ring, and four pre-shaped beams, as illustrated in **Figure** S7-a. By carefully selecting geometry parameters for the pre-shaped beam - specifically, a non-dimensional height, *H*/*L*, of 0.196 and a non-dimensional radius, *R*/*L*, of 0.41 - the module design achieves an equilibrium angle of 45°, as validated by the torque-angle curve obtained from FEA (**Figure** S7-b). The corresponding integrated system is designed with 8 elastic ports located at each 45° increment, with the equivalent circuitry diagram depicted in **Figure** S7-c and d. The initial and second stable mechanical configurations (denoted as “**+**” and “**ⅹ**”) correspond to the 0 and 1 state, respectively. In the initial state, the system establishes two connected electrical pathways and two disconnected electrical pathways. Upon a 45° rotation, the system transitions to the “**ⅹ**” state (1) with the buckled beams. The connection status of four electrical pathways reverses compared to the initial state. Consequently, these four electrical pathways function as two groups of NOT and Buffer logic elements, as illustrated in **Figure** S7-c and d. The module operates as a multiple-output rotary switch, selectively activating one of the two pathway groups. The proposed rotation-driven mechanism demonstrates its potential to simultaneously generate multiple logical outputs from a single mechanical input. Leveraging this mechanism allows us to realize the same combinational logic function with fewer mechanical computational elements, compared to other mechanisms with similar complexity which do not produce a complementary output. **Figure** S7-e illustrates the integration of the 45° rotation-driven bistable module with conductive materials (in orange), enabling it to function as a conductive mechanical bistable switch. To ensure proper operation, conductive materials are attached to the three rings along the *x*-direction on the front surface and the three rings along the *y*- direction on the back surface.


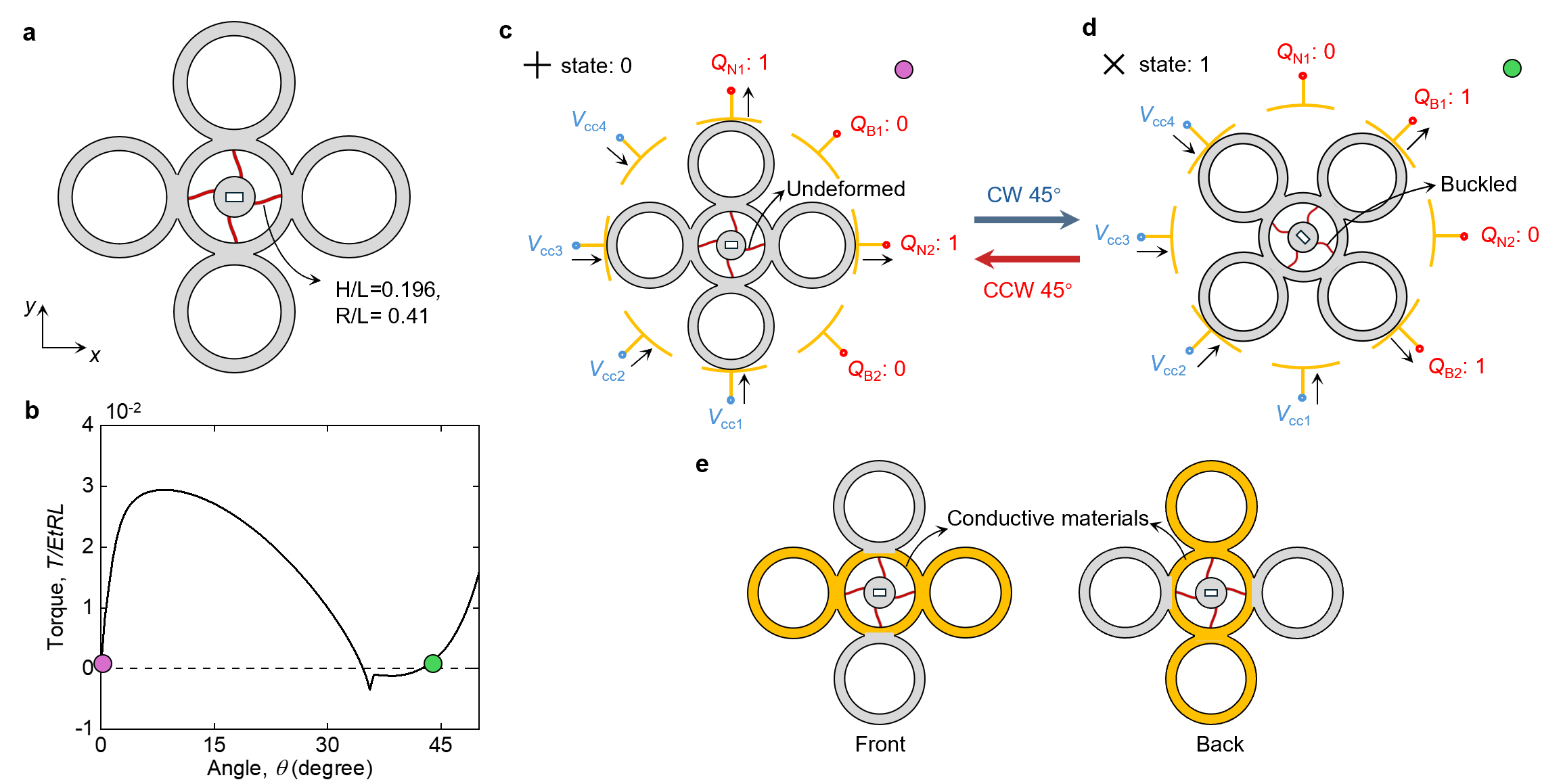


FIG. S7: (a) A 45° rotation-driven bistable module with non-dimensional geometry parameters: *H*/*L=* 0.196, and *R/L =* 0.41. (b) Non-dimensional torque-angle curve of a rotation-driven bistable module with an equilibrium angle of 45°. (c-d) Equivalent circuit diagrams of a 45° rotation-driven bistable building block with multiple NOT-Buffer outputs. (c: initial state, d: second stable state). (e) Integration method of the module with conductive materials (in orange).

**III Application****s**

**i)** *A four-bit FSM with reprogrammable transition rules*


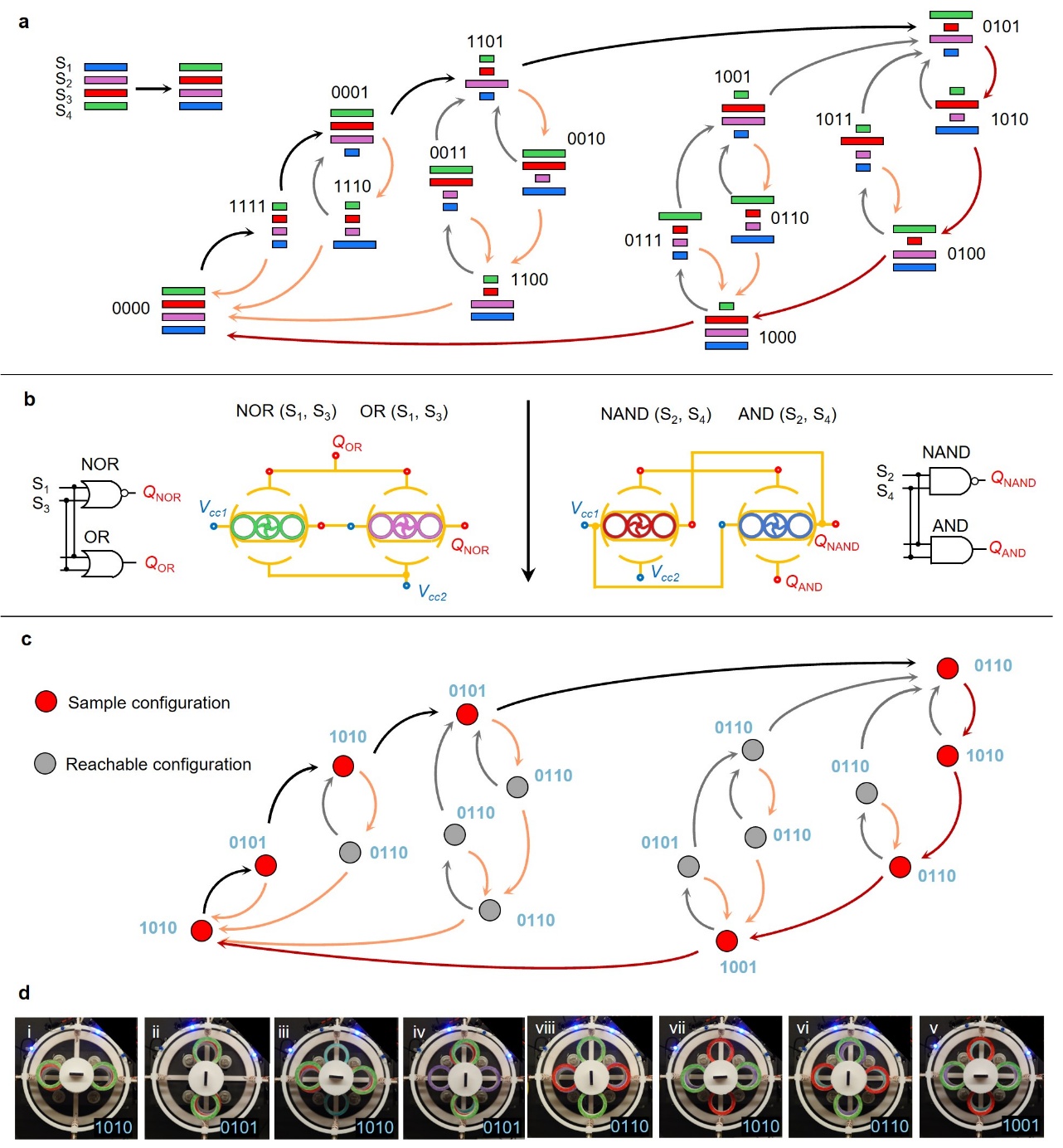


FIG. S8: A four-bit FSM with reprogrammable transition rules. (a)Transition graph of mechanical states with stacking order of decreasing thickness from top to bottom. (b) Electrical modular logic network delivering NOR (S1, S3), OR (S1, S3), NAND (S2, S4), and AND (S2, S4). (c) Transition graph of electrical outputs. (d) Experimental snapshots with multistable configurations and electrical outputs.

**ii)** *Four-bit encoding rules defining the trajectory of the car*

The real-time digital control is demonstrated using the four-bit FSM to drive a Mecanum-wheeled car (**Figure** 6E) along a pre-determined path. The four outputs of the system, which control the steering, are sent to a 433 MHz radio transmitter. These signals are received by a radio receiver and Raspberry Pi on the car and used to control the car’s motors from the signals generated by the system (**Figure** S9-a). The four bits control the power and direction of pairs of two wheels:

- Bit 1: Controls the start/stop state of Wheels 1 and 4.
- Bit 2: Controls the start/stop state of Wheels 2 and 3.
- Bit 3: Controls the forward/backward rotation of Wheels 1 and 4.
- Bit 4: Controls the forward/backward rotation of Wheels 2 and 3.

The four wheels are designated as Wheel 1, 2, 3, and 3 in clockwise order. Pair of wheels will not rotate when their “start/stop” bit (Bit 1 and Bit 2) is 0, regardless of the forward/backward bit state (Bit 3 and Bit 4). This encoding mechanism, illustrated in **Figure** S9-b and c, enables full omnidirectional motion control of the car (**Figure** S9-d). A Python script running on the Raspberry Pi reads the individual input bits from the radio receiver and generates the appropriate PWM signals to control the car’s motors. To illustrate the encoding rules, we provide an example of the car moving in the forward-left direction input in **Figure** S9-b. To move both forward and left, Wheels 1 and 4 remain still, while Wheels 2 and 3 rotate forwards. The resulting force vector, obtained from the superposition of the individual wheel forces, directs the car forwards and left.

For the radio transmitter and receiver hardware, we used the TE Connectivity / Linx Technologies EVAL-433-KH3 kit, which has battery-powered 433 MHz transmitter and receiver boards, and provides an 8-bit parallel interface for both input and output. We connected the 4 output wires from the system to 4 of the transmitter input bits and connected the same 4 receiver output bits to general-purpose I/O pins on the Raspberry Pi, for the Python script to read. The “voltage source” input of the material system comes from the transmitter’s battery. The receiver uses a 3V supply voltage, making it compatible with Raspberry Pi’s 3.3V digital inputs.


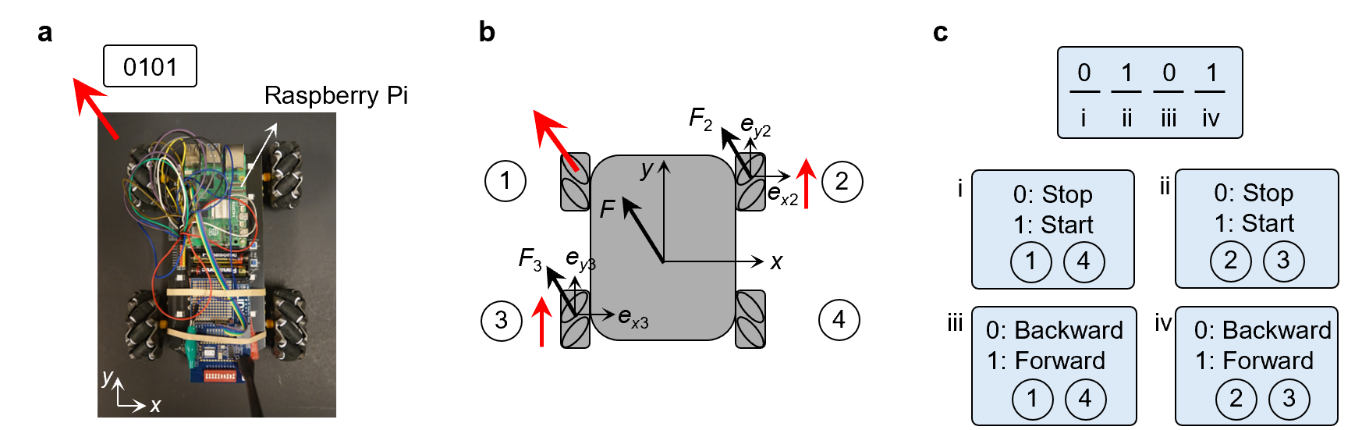


FIG. S9: (a) A Mecanum-wheeled car, equipped with a receiver and a Raspberry Pi, moves in the forward-left direction under the control of a four-bit signal (0101). (b) The car’s movement direction (large red arrow) is determined by the resultant force vector (large black arrow), generated through the superposition of the individual wheel forces (small black arrows). Each wheel’s force is controlled by each wheel’s rotation direction (small red arrows). (c) Four-bit encoding rules governing the movement of the car.

**IV Fabrication of the rotary FSM and torsional experimental tests**

**i)** *Fabrication of samples*

The four-layer electro-mechanical architecture is experimentally demonstrated in **Figure** S10-a, which comprises soft elements (bistable modules and elastic ports) and rigid elements (rigid base, rigid rotation element, and serial coupling elements), both 3D printed (QIDI, China). **Figures** S10-b and c show the geometry of the rotation elements and serially coupling elements. The soft modules were fabricated from white TPU filament, while the rigid elements from PLA filament. The 3D printing parameters for TPU are as follows: layer height of 0.16 mm, printing speed of 30 mm/s, infill density of 100%, and printing temperature of 210 °C. For PLA, the corresponding parameters are a layer height of 0.2 mm, printing speed of 60 mm/s, infill density of 100%, and printing temperature of 200 °C. To experimentally investigate the role of the in-plane thickness *t* on mechanical properties for given values of all the other geometric parameters, we conducted a series of torsion tests on bistable modules with varying thickness. The experimental results closely match the numerical result shown in **Figure** 1C. All experiments were repeated three times to provide statistical context.


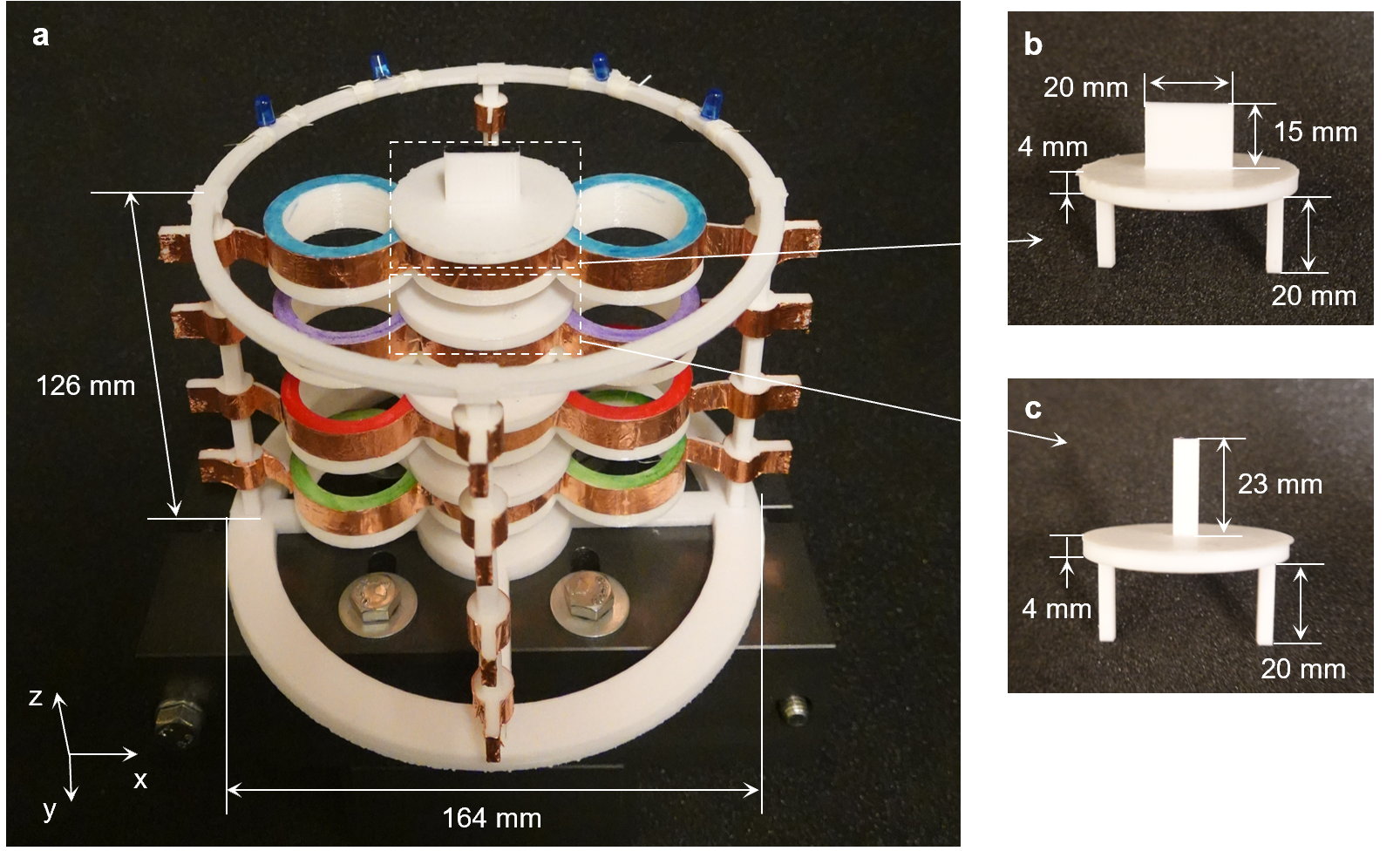


FIG. S10: (a) Experimental demonstration of the four-layer architecture. (b) Geometry of the rotation elements. (c) Geometry design of the serial coupling element.

**ii)** *Torsional tests*

The torque-angle curves of our experimental results were obtained through the torque testing apparatus, shown in **Figure** S11. The sample was mounted on a rigid support and connected to a rotary torque sensor via a rigid connection shaft. The other end of the rotary torque sensor was connected to a stepper motor with integrated driver and controller. Rigid bases beneath the rotary sensor and stepper motor ensured proper alignment of the rotation center among the sample, sensor and motor. The stepper motor’s embedded controller regulated the desired rotation speed at a 2 degree/second at room temperature, ensuring a quasi-static loading process. The applied torque and rotation angle were continuously monitored and recorded by the rotary torque sensor, with data transferred to a computer program to generate the torque-angle curves.


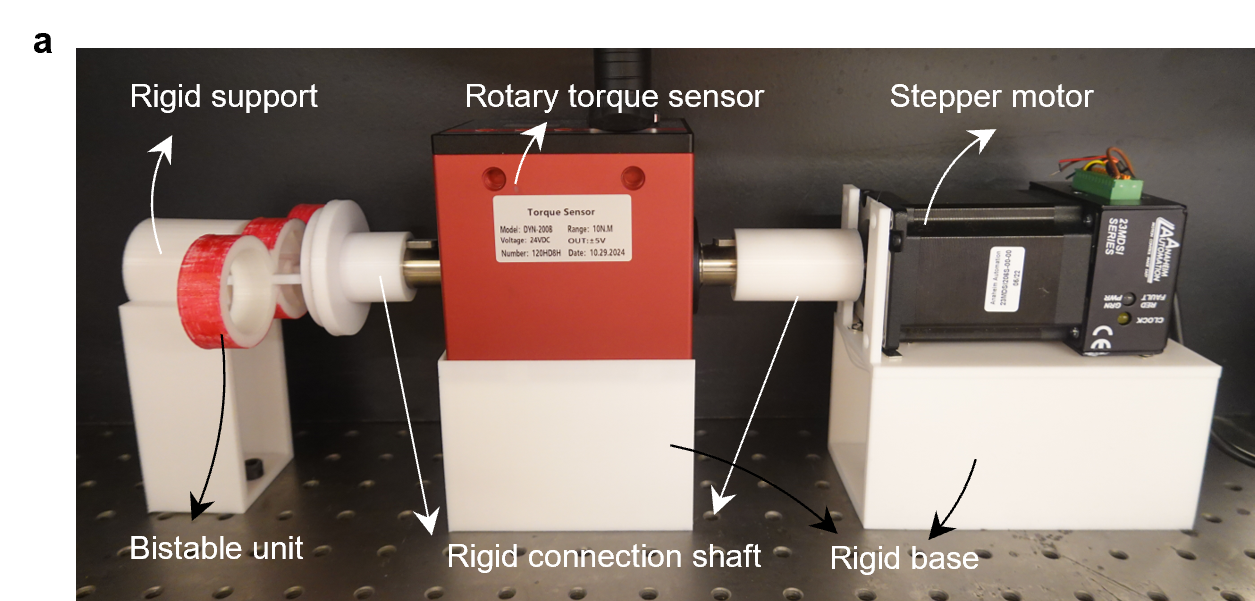


FIG. S11: Experimental setup for torsional tests.

**V Base material characterization**

To determine the material properties for the Finite Element analysis, we conducted uniaxial tension tests on five dog-bone specimens following ASTM D638 standards. The nominal stress-strain relationship is presented in **Figure** S12-a. The tensile tests were performed using a STEP Lab electrodynamic tester (STEP Engineering S.r.l., Resana, Treviso, Italy) with one load cell (AEP Transducers, Cognento, Italy) under displacement-control, as illustrated in **Figure** S12-b. The Young’s modulus of TPU was evaluated as the average slope of the stress-strain curve within the strain range of 0 to 0.01 [3]. The Poisson’s ratio was set as 0.47 [7], and the mass density was measured as 1170 kg·m^-3^.


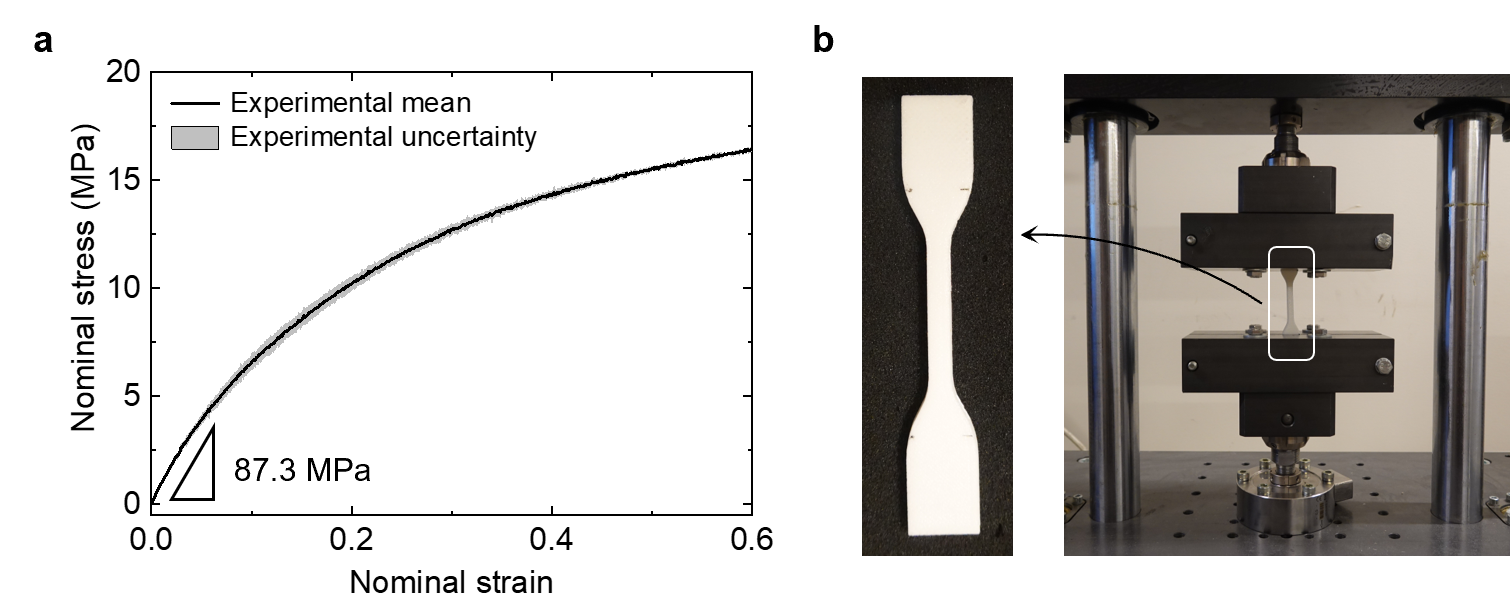


FIG. S12: (a) Nominal stress-strain relationship of the base material-White TPU. (b) Uniaxial tensile experimental tests performed on the Step tester.

**VI Supplementary Movies**

**Supplementary Movie S1.** Rotation-driven non-reciprocal mechanical memory.

**Supplementary Movie S2.** Rotation-driven sequential AND-NOR logic.

**Supplementary Movie S3.** Rotation-driven sequential OR-NAND logic.

**Supplementary Movie S4.** Rotation-driven sequential XNOR logic**.**

**Supplementary Movie S5.** Targeted trajectory control of a Mecanum-wheeled car.

**References**

1. Pan, D., et al., *Novel rotational motion actuated beam-type multistable metastructures.* Materials & Design, 2022. **224**: p. 111309.

2. Pan, D., et al., *The metastructures actuated by rotational motion with quasi-zero stiffness, negative stiffness, and bistability.* Thin-Walled Structures, 2025. **207**: p. 112700.

3. Yang, H. and L. Ma, *1D to 3D multi-stable architected materials with zero Poisson's ratio and controllable thermal expansion.* Materials & Design, 2020. **188**: p. 108430.

4. Wu, L. and D. Pasini, *In situ activation of snap‐through instability in multi‐response metamaterials through multistable topological transformation.* Advanced Materials, 2023. **35**(36): p. 2301109.

5. Udani, J.P. and A.F. Arrieta, *Programmable mechanical metastructures from locally bistable domes.* Extreme mechanics letters, 2021. **42**: p. 101081.

6. Sato, S., et al., *Dry sliding friction and Wear behavior of thermoplastic polyurethane against abrasive paper.* Biotribology, 2020. **23**: p. 100130.

7. Chen, B., et al., *Novel multifunctional negative stiffness mechanical metamaterial structure: tailored functions of multi-stable and compressive mono-stable.* Composites Part B: Engineering, 2021. **204**: p. 108501.
